# Systemic inflammation measured by erythrocyte sedimentation rate and cognitive function among young men in Sweden: A within-sibling analysis

**DOI:** 10.1177/00368504221145541

**Published:** 2023-01-30

**Authors:** Yin Xu, Ayako Hiyoshi, Katja Fall, Scott Montgomery

**Affiliations:** 1 Clinical Epidemiology and Biostatistics, School of Medical Sciences, Örebro University, Örebro, Sweden; 2 Department of Sociology and Psychology, School of Public Administration, 12530 Sichuan University, Chengdu, China; 3 Department of Public Health Sciences, Stockholm University, Stockholm, Sweden; 4 Department of Epidemiology and Public Health, 4919 University College London, London, UK; 5 Department of Integrative Epidemiology, Institute of Environmental Medicine, 27106 Karolinska Institutet, Stockholm, Sweden; 6 Clinical Epidemiology Division, Department of Medicine, Solna, Karolinska Institutet, Stockholm, Sweden

**Keywords:** Erythrocyte sedimentation rate, cognitive function, within-sibling analysis, late adolescence, Sweden

## Abstract

This study assesses the extent to which the association between erythrocyte sedimentation rate, a marker of inflammation, and cognitive function is explained by shared familial factors using within-sibling analyses. Men who were born in Sweden between 1950 and 1965 and recorded in the Swedish Military Conscription Register between 1969 and 1983 were included (*N* = 632,396). Erythrocyte sedimentation rate and cognitive function were measured at the conscription assessment (median age = 18.3 years, with a range from 15.5 to 28.5 years). Conventional linear regression and multilevel linear regression with a hybrid modeling approach were used, with the latter to obtain within-effect estimation in which unmeasured familial confounding shared by siblings was controlled for. We found that the association between erythrocyte sedimentation rate and cognitive function at conscription assessment was partly accounted for by, but remained independent of, shared familial factors.

## Introduction

Systemic inflammation, including low-grade inflammation, measured by elevated concentrations of inflammatory markers, including erythrocyte sedimentation rate (ESR), C-reactive protein (CRP), fibrinogen, and interleukin-6 (IL-6), has been associated with poorer cognitive function.^1,2^ Although this association may be bidirectional,^3–5^ a systematic review based on five prospective studies found that elevated concentrations of CRP at baseline was associated with poorer cognitive function measured at follow-up among elderly people.^1^ A meta-analysis based on four prospective or longitudinal studies also suggests that higher systemic levels of CRP were associated with an increased risk of cognitive decline among individuals without dementia (odds ratio = 1.27).^2^ Furthermore, animal studies also found that inflammation, including neuroinflammation, induced by lipopolysaccharide injections can lead to neuronal loss, microglia activation, and elevation in beta-amyloid peptide level, resulting in poorer cognitive function.^6,7^

It is possible that the association between markers of systemic inflammation and cognitive function may be partly explained by common genetic factors relevant to both cognitive function and systemic inflammation. A systematic review based on genetic association studies suggests that IL 1β single-nucleotide polymorphisms (SNPs), such as SNP rs16944, may be associated with both pro-inflammatory cytokines and memory performance, although these studies had methodological limitations, such as small sample size.^8^ Genome-wide association studies have further identified two genes involved in the immune response (*HS3ST4* and *SPOCK3*) associated with verbal declarative memory performance,^9^ and one gene involved in the expression of proinflammatory cytokines (*PDE7A*) associated with cognitive decline.^10^

Furthermore, the association between systemic inflammation and cognitive function may be partly explained by shared familial environmental factors. Studies found that the association between ESR and intelligence performance measured at ages 18–20 years attenuated after controlling for shared familial environmental factors, including household crowding, parental socioeconomic characteristics, and parental history of non-affective psychoses.^11,12^ Co-relative control analysis, which compares the effect size for the association between ESR and intelligence performance between the general population and pairs of relatives with different levels of relatedness (e.g. cousin, half-sibling, and full-sibling pairs), also suggested that this association was partly accounted for by familial factors.^11^

To the best of our knowledge, no prior research has examined whether the association between systemic inflammation, including low-grade inflammation and cognitive function in late adolescence or early adulthood is independent of shared familial genetic and environmental factors using sibling comparison analysis, which controls for unmeasured shared familial factors by comparing siblings discordant for cognitive function within a family. Therefore, we used a large general population-based birth cohort of Swedish men whose information on systemic inflammation and cognitive function were measured when they attended military conscription assessment.

## Methods

### Participants

**Figure 1.**
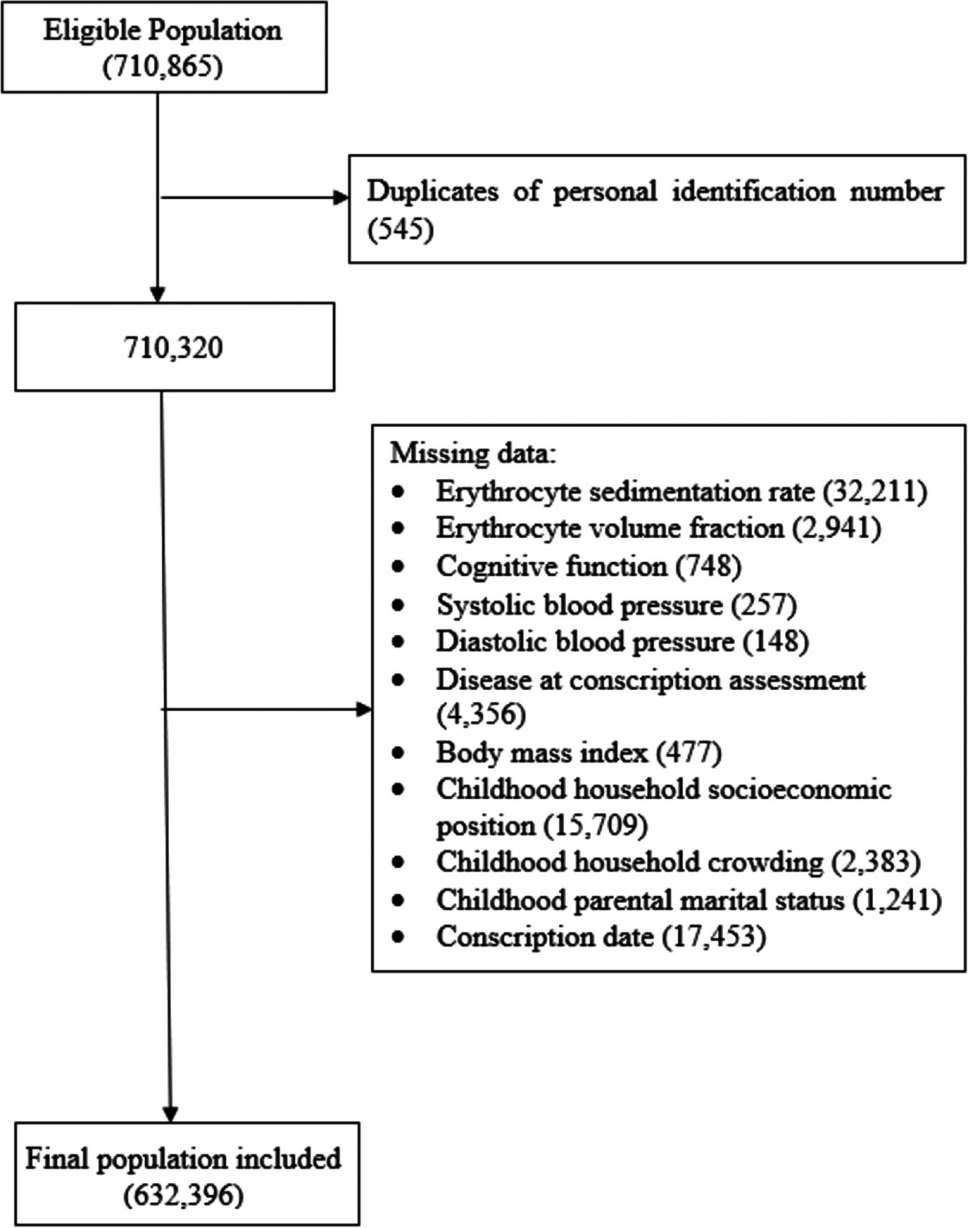
Flowchart of participant selection. Duplicates are due to the assignment of a previously deceased person's personal identification number to some immigrants to Sweden. Eligible population includes men who were born in Sweden between 1950 and 1965, who reached age 25 years in 1990, with at least one parent who was also alive in 1990 and were included in the Swedish Military Conscription Register between 1969 and 1983.

The cohort comprises all men who were born in Sweden between 1950 and 1965, who reached age 25 years in 1990, with at least one parent who was also alive in 1990 (to allow identification of first-degree relatives) and were included in the Swedish Military Conscription Register (to identify physical and psychological characteristics at conscription assessment) between 1969 and 1983 (only during this period, both cognitive function and ESR were collected). The unique individual Swedish personal identification number (converted into an anonymized identifier) was used for data linkage among the Swedish Total Population Register (to identify dates of birth, death, and migration, and cohort members’ biological parents), the Population and Housing Census data (to identify childhood parental marital status and socioeconomic characteristics), and the Conscription Register. All population-based registers used here have high validity and details about these registers have been described elsewhere.^13–15^ A total of 632,396 men were included (see Figure 1). The median age at conscription assessment was 18.3 years (interquartile range = 0.7), with a range from 15.5 to 28.5 years (only 18 men had an age<16 years at conscription assessment). The conscription rate for men was around 98% before 1995, as it was compulsory. Exemption from conscription assessment was most likely due to mental health disorders, severe chronic disease, or disability documented in a medical certificate.^16^

No formal power calculation was performed in advance since this study is based only on data from registries with a fixed sample size. However, it is possible to consider whether the study has the power to detect a meaningful effect. At the 5% level, the number of men included in the current study is much larger than the sample size required for the detection of a medium effect (Cohen's *f*^2^ = 0.15) with 90% power.

### Measures

#### Cognitive function

Cognitive function was assessed using two versions of the Swedish Enlistment Battery (SEB): SEB67 (from 1969 to 1979) and SEB80 (from 1980 to 1993). SEB67 and SEB80 consist of four subtests to measure visuospatial ability (Paper Form Board and Metal Folding tests were used in SEB67 and SEB80, respectively), verbal ability (Concept Discrimination and Synonyms tests were used in SEB67 in SEB80, respectively), ability to solve technical and physical problems (the Technical Comprehension test was used in both SEB67 and SEB80), and problem-solving, numerical, and logical abilities (the Instructions test was used in both SEB67 and SEB80).^17,18^ The Paper Form Board test involved judgments of which one out of four alternatives would correspond to the assembled object displayed in a set of disarranged parts. For the Metal Folding test, participants were required to find one three-dimensional object that agrees with a two-dimensional unfolded piece of metal among four three-dimensional alternatives. The Concept Discrimination test involved the identification of the word that did not fit in with the other five words. The Synonyms test involved the selection of the correct synonym for the target word among four alternatives. The Technical Comprehension test involved the selection of one out of three solutions to technical and physical problems. The Instructions test involved the selection of the answer that meets the conditions given by the instructions. The sum of four subtests was transformed into a standardized 9-point scale based on the sample distribution for each conscription year, with a higher score indicating better general ability. Cognitive function at ages 18–20 years measured using SEB67 was associated with subsequent income and socioeconomic position at age 40 years.^19^ A study also reported high internal consistency for SEB80.^18^ Thus, both SEB67 and SEB80 are acceptable measures of general ability.^17^ The descriptive statistics of cognitive function across the year at conscription have been described elsewhere.^20^

#### ESR and erythrocyte volume fraction

Venous samples taken at conscription assessment were used for the analyses of ESR and erythrocyte volume fraction (EVF). ESR (mm/h), a nonspecific marker for inflammation, was assessed using the Westergren method and defined as the distance that a column of anticoagulated blood falls for an hour. EVF (%) was assessed using the microhematocrit method and defined as the proportion of blood occupied by red blood cells. ESR < 1 or > 98 mm/h and EVF < 0.20 or > 0.75 were treated as non-valid values. Since EVF influences ESR, EVF was included in the linear regression models as an adjustment for ESR.

#### Potential confounding factors

These included physical and health characteristics at conscription assessment (systolic and diastolic blood pressure, body mass index (BMI), disease diagnoses, and age at conscription), childhood socioeconomic characteristics (household crowding and household socioeconomic position between birth and age 10 years), childhood parental marital status (married or unmarried), and an indicator of the SEB version used at conscription assessment (SEB67 or SEB80). Systolic and diastolic blood pressure (mmHg) of the right arm was measured using sphygmomanometers after 5–10 min of rest in the supine position, in accordance with a written protocol.^21^ BMI (kg/m^2^) was calculated using weight in light clothing (kg) divided by height (m) without shoes squared and defined as underweight (15 to <18.5), normal weight (18.5 to < 25), overweight (25 to < 30), and obesity (≥ 30). Systolic blood pressure <50 or >230 mmHg, diastolic blood pressure <30 or >135 mmHg, or BMI <15 kg/m^2^ was treated as non-valid values. Disease diagnoses at conscription assessment after medical examinations are supplied as a summary score with 10 levels, with a lower score, in general, indicating severer health problems. This was grouped into no diagnosis, no serious diagnosis, fairly significant disease, and significant health problems. Household crowding was derived using the number of residents divided by the number of habitable rooms within the same household (kitchen not included) and classified into four categories: ≤1, >1 to 2, >2 to 3, and >3 persons per room. Household socioeconomic position was derived using the occupation of the head of household and classified into six groups: manual workers, intermediate non-manual employees, farm owners/managers, self-employed professionals, self-employed other than professions, and unemployed/not in the labor market. Parental marital status was classified as married and unmarried, with the latter including parents who were single, divorced, or widowed.

### Statistical analysis

Several regression models were used to test the association of ESR with cognitive function at conscription assessment. First, multivariable linear regression was used with adjustment for EVF, childhood socioeconomic characteristics, physical and health characteristics at conscription assessment, childhood parental marital status, and the SEB version used at conscription assessment. Fractional polynomials suggested that ESR, EVF, systolic blood pressure, and age at conscription assessment were non-linearly associated with cognitive function at conscription assessment. Accordingly, the transformations suggested by fractional polynomial for ESR, EVF, systolic blood pressure, and age at conscription assessment were used in all regression models, (ESR/10)^0.5^, (EVF/10)^3^, (systolic blood pressure/100)^−2^, and (age at conscription assessment/10)^−2^, respectively. Standardized residuals were normally distributed based on a quantile–quantile plot. Homoscedasticity was also not violated based on a scatter plot of predicted values and standardized residuals. Huber-White standard errors were used to account for clustering within families.

To adjust for unmeasured shared familial genetic and environmental factors, a multilevel linear regression was used with a hybrid modeling approach, which estimated both within- and between-effects of ESR on cognitive function, independent from each other, in a single model.^22^ Within-effect estimates represent the association of ESR with cognitive function based on the comparison with siblings within the same family, which controls for familial genetic and environmental factors commonly shared by siblings. Between-effect estimates represent the association of ESR with cognitive function comparing unrelated men. The *Wald* test was used to determine whether the within-effect is different from the between-effect estimate. If the coefficient for ESR obtained from the within-effect estimate was lower than that obtained from the between-effect estimate, this suggests that the association of ESR with cognitive function was partially explained by the unmeasured shared familial genetic and environmental factors. The mother's Swedish personal identification number (converted into an anonymized identifier) was used to identify the observations belonging to the same family, and when it was missing, the father's Swedish identification number was used.

To obtain the overall association between ESR and cognitive function, a multilevel linear regression model with a random intercept was also used. The coefficient in this model is the weighted average of the within- and between-effects by the inverse of its variance. The likelihood ratio test suggested that the model fit was not changed with or without including a random slope for ESR, *x*^2^(2) = 3.21, *p* = 0.201. Thus, a random slope was not included in the model. Variables included in the multilevel linear regression model with a random intercept and variables transformation were the same as for the multilevel linear regression with a hybrid modeling approach.

A total of three sensitivity analyses using multilevel linear regression with a hybrid modeling approach were performed. In the first sensitivity analysis, outliers (*n* = 60,235) which were defined as with an ESR greater than (median + 3*median absolute deviation) or lower than (median −3*median absolute deviation) were excluded to examine the influence of outliers.^23^ In the second sensitivity analysis, families with only one child were excluded to examine the influence of sibship size. In the third sensitivity analysis, only men without any disease diagnoses at the conscription assessment were included to exclude the potential influence of any disease diagnosed at the conscription assessment. All analyses were performed in Stata 16.0.

## Results

A total of 632,396 men from 499,776 families were included (the average number of male offspring in a family is 1.27). The mean cognitive function score was 5.20 (SD = 1.94). The median ESR value was 2 mm/h. Within-effects estimates indicated a more crowded household in childhood (>1 person per one room), underweight, overweight, or obesity at conscription assessment, having any disease diagnosis at conscription assessment, and younger age at conscription assessment were associated with lower cognitive function score (see Table 1).

**Table 1.** Characteristics of participants in the cohort and adjusted coefficients for cognitive function at conscription assessment.

Variables	Descriptive information	Linear regression	Multilevel linear regression with hybrid modeling approach		Multilevel linear regression with random intercept
			Within-effect	Between-effect	Overall effect
Erythrocyte sedimentation rate (mm/h)					
Median (interquartile range)	2.00 (2.00)	−0.18 (−0.21, −0.16)	−0.07 (−0.11, −0.03)	−0.20 (−0.22, −0.17)	−0.17 (−0.19, −0.14)
Erythrocyte volume fraction (%)					
Mean (SD)	46.39 (2.44)	−0.00 (−0.00, −0.00)	−0.00 (−0.00, −0.00)	−0.00 (−0.00, −0.00)	−0.00 (−0.00, −0.00)
Systolic blood pressure (mmHg)	128.35 (11.07)	0.13 (0.09, 0.18)	0.16 (0.08, 0.24)	0.13 (0.08, 0.17)	0.13 (0.09, 0.17)
Diastolic blood pressure (mmHg)	68.89 (9.69)	−0.00 (−0.00, −0.00)	−0.00 (−0.00, 0.00)	−0.00 (−0.00, −0.00)	−0.00 (−0.00, −0.00)
Age at conscription assessment (years)	18.34 (0.63)	−9.71 (−9.96, −9.46)	−7.75 (−8.19, −7.30)	−10.12 (−10.40, −9.83)	−9.37 (−9.61, −9.13)
Household socioeconomic position, *n* (%)					
Manual workers	293,885 (46.47)	Reference group	Reference group	Reference group	Reference group
Intermediate non-manual employees	204,863 (32.39)	0.92 (0.91, 0.93)	0.05 (0.00, 0.09)	0.92 (0.91, 0.94)	0.89 (0.88, 0.90)
Farm owners/managers	49,358 (7.80)	0.11 (0.09, 0.13)	−0.09 (−0.16, −0.01)	0.11 (0.09, 0.13)	0.09 (0.07, 0.10)
Self-employed (other than professions)	44,652 (7.06)	0.24 (0.22, 0.26)	0.01 (−0.06, 0.07)	0.25 (0.23, 0.27)	0.23 (0.21, 0.25)
Self-employed professions	20,792 (3.29)	1.07 (1.05, 1.10)	0.03 (−0.06, 0.12)	1.08 (1.05, 1.11)	1.04 (1.01, 1.07)
Unemployed/not in the labor market	18,846 (2.98)	0.48 (0.45, 0.51)	0.00 (−0.09, 0.09)	0.50 (0.47, 0.53)	0.47 (0.44, 0.50)
Household crowding, *n* (%)					
≤ 1	141,291 (22.34)	Reference group	Reference group	Reference group	Reference group
(1, 2]	372,487 (58.90)	−0.42 (−0.43, −0.41)	−0.05 (−0.08, −0.01)	−0.42 (−0.43, −0.40)	−0.40 (−0.41, −0.39)
(2, 3]	85,407 (13.51)	−0.78 (−0.80, −0.77)	−0.05 (−0.10, −0.00)	−0.78 (−0.80, −0.76)	−0.73 (−0.75, −0.72)
> 3	33,211 (5.25)	−0.92 (−0.95, −0.90)	−0.11 (−0.18, −0.04)	−0.91 (−0.94, −0.89)	−0.87 (−0.89, −0.84)
Body mass index, *n* (%)					
Underweight	63,399 (10.03)	−0.08 (−0.09, −0.06)	−0.06 (−0.08, −0.03)	−0.08 (−0.09, −0.06)	−0.07 (−0.08, −0.05)
Normal weight	514,733 (81.39)	Reference group	Reference group	Reference group	Reference group
Overweight	46,794 (7.40)	−0.27 (−0.29, −0.25)	−0.14 (−0.17, −0.11)	−0.29 (−0.31, −0.27)	−0.25 (−0.27, −0.24)
Obese	7470 (1.18)	−0.43 (−0.47, −0.39)	−0.29 (−0.36, −0.21)	−0.44 (−0.49, −0.40)	−0.41 (−0.45, −0.37)
Disease diagnoses at conscription, *n* (%)					
No diagnosis	308,793 (48.83)	Reference group	Reference group	Reference group	Reference group
No Serious diagnosis	238,487 (37.71)	−0.14 (−0.15, −0.13)	−0.11 (−0.12, −0.09)	−0.15 (−0.16, −0.14)	−0.14 (−0.15, −0.13)
Fairly significant disease	44,095 (6.97)	−0.45 (−0.47, −0.43)	−0.32 (−0.35, −0.29)	−0.46 (−0.48, −0.44)	−0.43 (−0.44, −0.41)
Significant health problem	41,021 (6.49)	−1.22 (−1.24, −1.20)	−0.84 (−0.88, −0.81)	−1.27 (−1.29, −1.24)	−1.15 (−1.17, −1.13)
Swedish Enlistment Battery, *n* (%)					
Swedish Enlist Battery 67	437,958 (69.25)	Reference group	Reference group	Reference group	Reference group
Swedish Enlist Battery 80	194,438 (30.75)	−0.12 (−0.13, −0.11)	−0.24 (−0.26, −0.22)	−0.09 (−0.10, −0.07)	−0.14 (−0.15, −0.13)
Childhood parental marital status, *n* (%)					
Unmarried	39,220 (6.20)	−0.48 (−0.50, −0.46)	−0.03 (−0.11, 0.04)	−0.50 (−0.52, −0.48)	−0.47 (−0.49, −0.45)
Married	593,176 (93.80)	Reference group	Reference group	Reference group	Reference group

*Note*. Coefficient and 95% confidence intervals from multivariable linear regression and multivariable multilevel linear regression, controlling for all variables listed in the Table 1, were reported here. The within- and between-estimates from multilevel linear regression with hybrid modeling approach were obtained from a single model. Fractional polynomial suggested that erythrocyte sedimentation rate, erythrocyte volume fraction, systolic blood pressure, and age at conscription assessment were non-linearly associated with cognitive function. Accordingly, the transformation suggested by fractional polynomial for erythrocyte sedimentation rate, erythrocyte volume fraction, systolic blood pressure, and age at conscription assessment was used in the analyses, (erythrocyte sedimentation rate/10)^0.5^, (erythrocyte volume fraction/10)^3^, (systolic blood pressure/100)^−2^, and (age at conscription assessment/10)^−2^, respectively.

**Figure 2.**
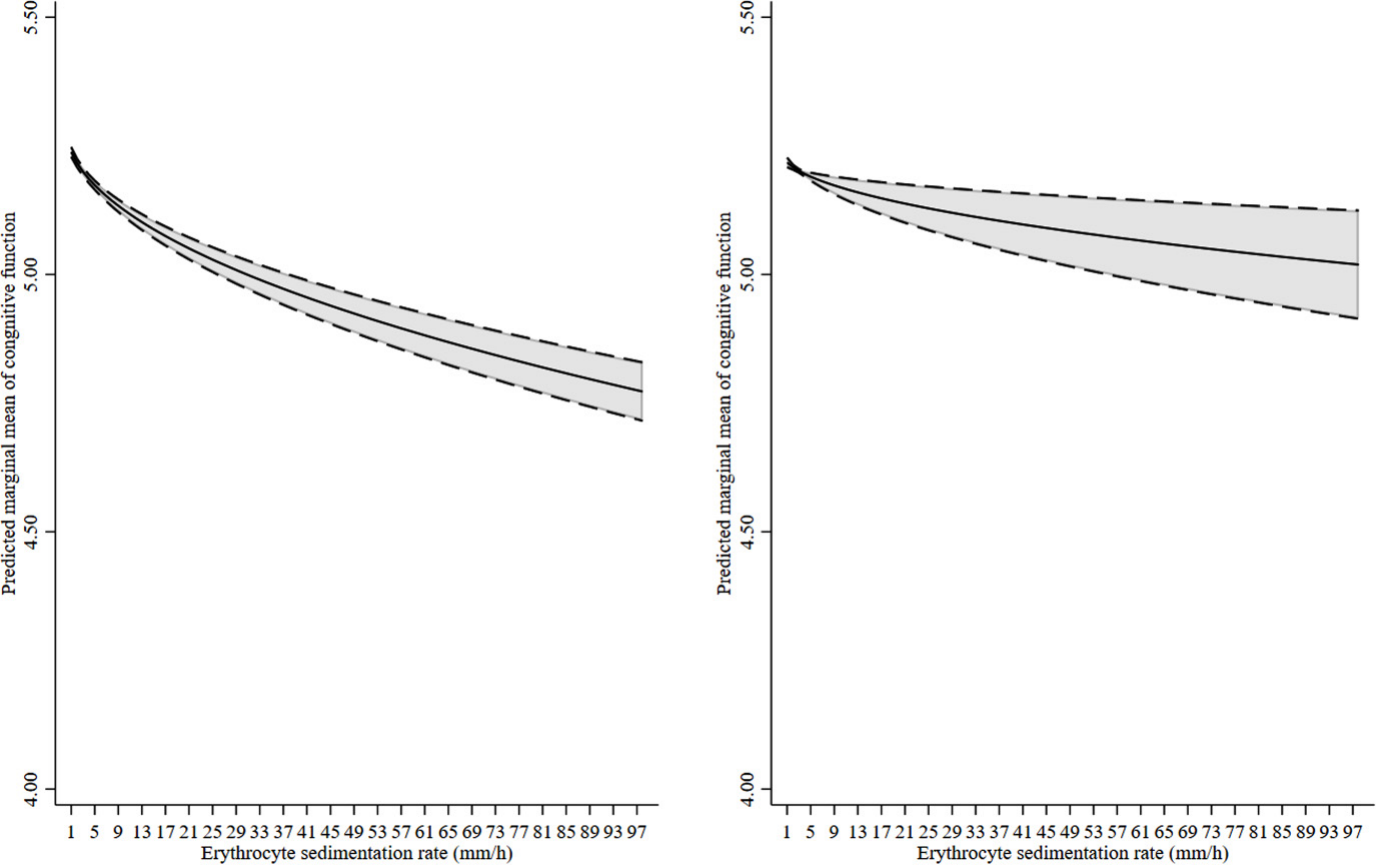
The predicted marginal mean of cognitive function with random intercept and 95% confidence interval stratified by erythrocyte sedimentation rate across the entire range. The overall association between erythrocyte sedimentation rate and cognitive function, controlling for family clustering in the estimation of 95% confidence interval, from multivariable multilevel linear regression with random intercept were plotted in the left panel. The within-effect estimates for erythrocyte sedimentation rate (ESR) were plotted in the right panel.

Higher ESR was associated with poorer cognitive function at conscription assessment in the univariate linear regression (with the adjustment only for EVF): coefficient (*β*) = −0.33, 95% confidence interval (CI) = [−0.35, −0.30], *p *< 0.001. This association decreased in magnitude but remained statistically significant after further adjustment for childhood socioeconomic characteristics, physical characteristics at conscription assessment, childhood parental marital status, and SEB version used at conscription assessment: *β* = −0.18, 95% CI = [−0.21, −0.16], *p *< 0.001. The within-effect for ESR, controlling for unmeasured shared familial factors (within-sibling analysis), showed that this association further attenuated but remained statistically significant, *β* = −0.07, 95% CI = [−0.11, −0.03], *p* = 0.001. The within-effect for ESR was statistically significantly smaller compared with the between-effect for ESR, *x*^2^(1) = 26.68, *p *< 0.001, indicating the influences of unmeasured shared familial factors on the association of cognitive function with ESR. The multilevel linear regression model with a random intercept showed that the overall association between ESR and cognitive function at conscription assessment followed a non-linear and dose-dependent pattern (see Figure 2).

The first sensitivity analysis showed that outliers did not affect the effect size of the association between ESR and cognitive function at conscription assessment, *β* = −0.11, 95% CI = [−0.18, −0.04], *p* = 0.003 and *β* = −0.20, 95% CI = [−0.24, −0.16], *p *< 0.001 for within- and between-effects, respectively. The second sensitivity analysis showed no substantial differences in *β* between analyses including and excluding families with only one child, *β* = −0.07, 95% CI = [−0.11, −0.03], *p* = 0.001 and *β* = −0.28, 95% CI = [−0.34, −0.22], *p *< 0.001 for within- and between-effects, respectively. The third sensitivity analysis showed that this association remained statistically significant when men with any disease diagnosis at the conscription assessment were excluded, *β* = −0.13, 95% CI = [−0.21, −0.06], *p* = 0.001 and *β* = −0.16, 95% CI = [−0.20, −0.13], *p *< 0.001 for within- and between-effects, respectively.

## Discussion

We found that higher ESR, a marker of systemic inflammation, was associated with poorer cognitive function at conscription assessment in late adolescence, even where there was only low-grade inflammation. This association followed a dose-dependent pattern and was independent of physical and health characteristics at conscription assessment and measured and unmeasured shared familial genetic and environmental factors which were controlled for using within-effect estimates in a multilevel linear regression with a hybrid modeling approach. The familial factors accounted for a notable proportion of the overall association. We also found that contemporaneous diagnoses did not explain the association between ESR and cognitive function at conscription assessment since the magnitude of this association was similar between analyses including and excluding men with any disease diagnoses at the conscription assessment.

Several mechanisms may help explain the association between ESR and cognitive function. Shared environmental factors appear one of the common causes associated with both systemic inflammation and poorer cognitive function. Prolonged exposure to shared adverse early familial life factors, including socioeconomic disadvantage, dysfunctional households, and maltreatment, could affect brain regions with prolonged developmental trajectories (e.g. decreases in cerebral volumes and corpus callosum size, reduced hemispheric integration, and reduced hippocampal volume) which may contribute to the deficits in cognitive function.^24^ Prolonged exposure to shared adverse early familial life factors could also lead to poorer health behavior, or cause dysfunction of cellular immunity and the hypothalamic–pituitary–adrenal axis, which increases the risk for systemic inflammation.^25,26^ For example, prolonged exposure to shared adverse early familial life factors could lead to change in cortisol levels or diurnal cortisol slope, and alteration in the expression and DNA methylation of genes associated with immune dysregulation (e.g. Toll-like receptor 4 mRNA^25^). Common genetic factors that load simultaneously on both systemic inflammation and cognitive function may be another shared familial factor, such as the *HS3ST4* and *SPOCK3* genes, as found in one genome-wide association study.^9^

Although the association between ESR and cognitive function was attenuated after controlling for shared familial factors using within-effect estimation, this association remained statistically significant, indicating a possibly small but non-negligible influence of ESR on cognition. This is consistent with animal models indicating inflammation being a risk factor for poorer cognitive function.^6,7^ Systemic inflammation may lead to adverse changes in brain morphology, including reduced cortical gray and white matter, and hippocampal volume, resulting in poorer cognitive function.^27^ Peripheral inflammatory markers (e.g. IL-6) can also cross the blood–brain barrier which results in neurodegeneration and a decline in cognitive function.^28,29^ This study could not identify the cause of the raised inflammation. One possible explanation is that prodromal activity of undiagnosed conditions such as Crohn's disease increase ESR while also resulting in prolonged fatigue and other exposures that are not conducive to optimal cognitive performance.^30^ This explanation and direct adverse consequences of raised inflammation on cognitive function are not mutually exclusive, although the frequency of such diseases in late adolescence, when most of the men in this study were measured ESR, is low. Alternatively, it may be that poorer cognitive function may lead to higher ESR as poorer cognitive function in childhood has been associated with engagement in unhealthy behavior such as less physical activity, smoking, and poorer dietary choices,^31^ increasing the risk of systemic inflammation by late adolescence.^32^ This is consistent with the observation that the association between ESR and cognitive function attenuated when conventional models were compared with the within-sibling analysis, as such behavior tends to cluster within families, although the possibility of a direct influence also exists as the association was not eliminated entirely in the sibling comparison models. Nonetheless, we cannot determine whether raised ESR precedes poorer cognitive function.

Our sibling comparison analysis also demonstrated that living in a more crowded household in childhood, and being underweight, overweight, or obese at conscription assessment was also associated with poorer cognitive function. As discussed above, socioeconomic disadvantage in childhood could affect brain regions over prolonged developmental trajectories (e.g. decreases in cerebral volumes), causing deficits in cognitive function.^24^ We could not determine the temporal pattern of association between BMI and cognitive function, but it is possible that poorer cognitive performance was associated with less physical activity and poorer dietary choices,^31^ increasing the risk of being overweight or obese.^33^ Alternatively, obesity may increase the risk of systemic inflammation,^34^ causing adverse changes in brain morphology,^27^ and resulting in poorer cognitive function. Nonetheless, the association between higher ESR and poorer cognitive function was independent of BMI.

The key strengths of the present study include the use of a large sample of Swedish men from a general population-based birth cohort with the high level of statistical power needed for within-sibling analysis. ESR and cognitive function were collected objectively at the conscription assessment which eliminates concerns about recall bias. In addition to a number of measured confounding factors, unmeasured shared familial factors were controlled for using within-effect estimates. Sensitivity analysis also found that the association between systemic inflammation and cognitive function at conscription assessment was independent of any diseases diagnosed at the conscription assessment. Nevertheless, several potential limitations are noteworthy. We cannot determine whether raised ESR precedes lower cognitive function. ESR was the only available measure of inflammation, and other markers could shed further light on the association, such as IL-6. Finally, only men could be included as the vast majority invited to the compulsory conscription assessment at this time: the results may not be generalizable to women.

In summary, higher ESR, indicating systemic inflammation, was associated with poorer cognitive function in young men. This was independent of measured and unmeasured shared familial factors (although familial factors appear to explain a notable proportion of the association), indicating that some of the relevant pathways may operate at the level of the individual.
